# The effect of resistance training interventions on weight status in youth: a meta-analysis

**DOI:** 10.1186/s40798-018-0154-z

**Published:** 2018-08-20

**Authors:** Helen Collins, Samantha Fawkner, Josephine N. Booth, Audrey Duncan

**Affiliations:** 1 Institute of Sport and Exercise, University of Dundee, Old Hawkhill, Dundee, DD14HN UK; 2 Physical Activity and Health Research Centre, University of Edinburgh, Edinburgh, UK; 3 Moray House School of Education, University of Edinburgh, Edinburgh, UK

**Keywords:** Resistance-training, Children, Adolescents, Obesity, Strength, Weight

## Abstract

**Background:**

There has been a rise in research into obesity prevention and treatment programmes in youth, including the effectiveness of resistance-based exercise. The purpose of this meta-analysis was to examine the effect of resistance training interventions on weight status in youth.

**Methods:**

Meta-analysis followed the Preferred Reporting Items for Systematic Reviews and Meta-Analyses guidelines and was registered on PROSPERO (registration number CRD42016038365). Eligible studies were from English language peer-reviewed published articles. Searches were conducted in seven databases between May 2016 and June 2017. Studies were included that examined the effect of resistance training on weight status in youth, with participants of school age (5–18 years).

**Results:**

There were 24 complete sets of data from 18 controlled trials (CTs) which explored 8 outcomes related to weight status. Significant, small effect sizes were identified for body fat% (Hedges’ *g* = 0.215, 95% CI 0.059 to 0.371, *P* = 0.007) and skinfolds (Hedges’ *g* = 0.274, 95% CI 0.066 to 0.483, *P* = 0.01). Effect sizes were not significant for: body mass (Hedges’ *g* = 0.043, 95% CI − 0.103 to 0.189, *P* = 0.564), body mass index (Hedges’ *g* = 0.024, 95% CI − 0.205 to 0.253, *P* = 0.838), fat-free mass (Hedges’ *g* = 0.073, 95% CI − 0.169 to 0.316, *P* = 0.554), fat mass (Hedges’ *g* = 0.180, 95% CI − 0.090 to 0.451, *P* = 0.192), lean mass (Hedges’ *g* = 0.089, 95% CI − 0.122 to 0.301, *P* = 0.408) or waist circumference (Hedges’ *g* = 0.209, 95% CI − 0.075 to 0.494, *P* = 0.149).

**Conclusions:**

The results of this meta-analysis suggest that an isolated resistance training intervention may have an effect on weight status in youth. Overall, more quality research should be undertaken to investigate the impact of resistance training in youth as it could have a role to play in the treatment and prevention of obesity.

## Key points

- Physical activity guidelines and position statements emphasise the importance of ‘activity to strengthen muscle and bone’ and research suggests that resistance training might have an impact on weight status in youth.
- This meta-analysis found that resistance training has a positive effect on body fat percentage and skinfolds in youth.
- Further research is required to investigate the role resistance training may play in the treatment and prevention of obesity.

## Background

Obesity is a worldwide concern. In 2016, more than 1.9 billion adults were classified as overweight, 13% were obese and 41 million children aged under 5 were overweight or obese [1]. Childhood obesity is a critical public health threat as the prevalence of obesity amongst youth continues to increase worldwide, and there is the risk of developing obesity-related diseases at an increasingly younger age [2]. Prevention and treatment programmes suitable for youth have been developed for which physical activity is an integral component [3].

The current guidelines for children aged 5–18 recommend 60 min of daily physical activity (PA), and minimising the time spent sitting each day. They also recommend activity that strengthens muscle and bone, at least 3 days a week [3, 4]. However, despite these guidelines, one of the more recent global surveillance studies, the Health Behaviour in School-aged Children survey [5], reported that across Europe, less than 50% of young people were meeting the current PA guidelines. Additionally, the survey demonstrated a decline in PA levels with age; 25% of 11 years olds met the recommendations, compared to just 16% of 15 year olds [5] which indicates that as children advance to adolescence, sedentary behaviour becomes more common.

A growing volume of studies have now been published that seek to examine the effectiveness of PA interventions designed to combat these low levels [6]. However, in a systematic review of 57 studies that investigated PA interventions in children and adolescents, very few studies that were included examined interventions which addressed compliance with the muscle and bone component of the guidelines [6].

The benefits of resistance training (RT) in youth are well documented, and key organisations (NSCA, UKSCA and BASES) have developed position statements in support of this [7–9]. A benefit identified in these position statements is the positive effect of RT on weight status, although the evidence to support these statements is not strong. In fact, the majority of research investigates multi-component interventions with a focus on overweight/obese youths [10–12].

A systematic review in 2011 identified that very few randomised control trials have examined the effects of RT alone on body composition in overweight or obese adolescents [10]. From just seven studies included in this review that focused on RT alone, the authors reported inconclusive results and the recommendation was for more studies to be conducted that concentrate solely on RT interventions [10]. Also examining the effect of RT alone on overweight or obese children, a further review found only six studies met the inclusion criteria [11]. Three out of the six studies showed a significant decrease in percentage fat and a significant increase in fat free mass, although none of the studies found a decrease in total fat mass. Additionally, all studies reported an increase in body weight. Again, the findings were inconclusive, but they identified there was a lack of randomised control trials (only two studies) and the studies had low sample sizes [11]. In a systematic review and meta-analysis, it was found that interventions that included a RT component had a very small to small effect on body composition in overweight and obese youth [12]. Studies that included interventions that were RT alone showed similar effects to those studies that included resistance plus an aerobic or dietary component. However, across studies, there was large variation in intervention content and assessment methods [12].

The studies included in these reviews involved only overweight and obese participants and therefore it could be argued that they are focused on the treatment of obesity rather than prevention. However, it has been suggested that early prevention holds better than treatment with ‘primordial prevention’ being the first of three levels of obesity prevention [13]. This emphasises the prevention of risk factors to maintain a healthy weight throughout childhood and into the teens [13].

There has been one systematic review to date with emphasis on the impact of RT on the weight status of both healthy weight and overweight/obese youth [14]. This review included studies using a variety of assessment tools and also studies that combined aerobic training and dietary interventions, however, there were still only 12 studies included in total. Investigating the impact of RT on body composition, they identified that due to limitations in methodologies it was difficult to reach conclusions about the isolated role of RT [14].

Since the focus of the published reviews has been on obese and overweight youth, or on multicomponent interventions, the isolated role of RT on weight status in youth is not currently known. Further to this, the only review to focus on both healthy weight and overweight/obese youth to date is a decade old. Therefore, the purpose of this review was to systematically examine the impact of resistance training interventions on weight status in youth.

## Methods

**Fig. 1:**
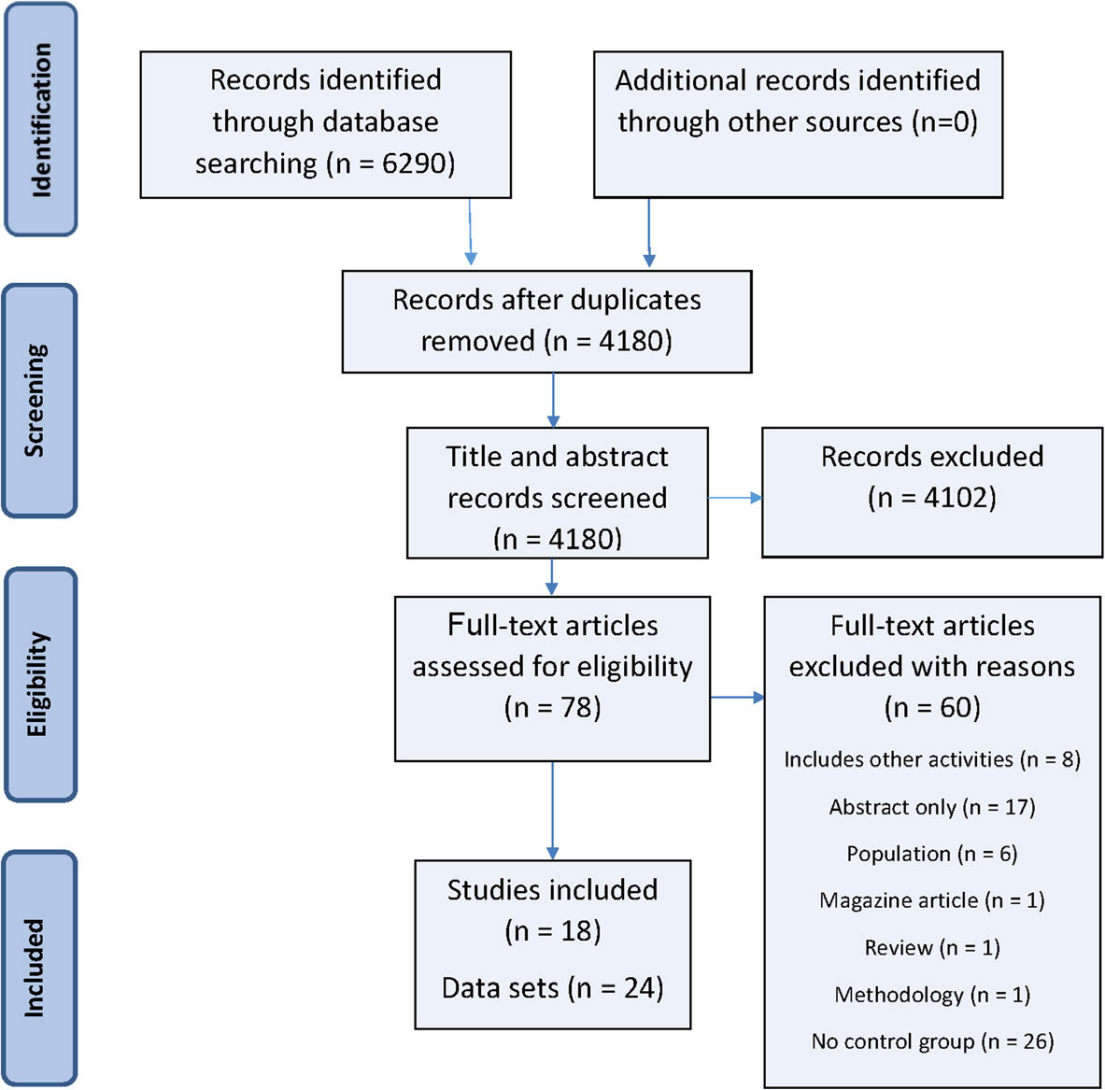
PRISMA flow diagram of systematic search and included studies

The search strategy and inclusion criteria were specified and documented in advance on PROSPERO (number CRD42016038365). The conduct and reporting of this review adhered to the guidelines outlined in the PRISMA statement [15]. The PRISMA flow diagram detailing the systematic search and included studies is shown in Fig. 1.

### Search strategy

Electronic literature databases were searched from the year of their inception up to and including June 2017. These were PubMed, MEDLINE, ERIC, PsycINFO, Embase, Sport Discus and Scopus. Relevant references from published literature were followed up and included when they met the inclusion criteria and literature not identified in the electronic searches was sourced. ResearchGate was used to identify research papers written by key researchers in the field. Additionally, these researchers were contacted regarding any literature not yet published and the authors of this review searched their personal libraries.

The search terms were related to weight status, youths and RT (Table 1). The Boolean operator “AND” was used between categories, and the phrase “OR” was used within categories.  The search strategy was adapted for each database, and searches were logged.

**Table 1:** Systematic review categories and search terms

Target population	Resistance training	Weight status
Youth^a^, young, child^a^, teen^a^, adol^a^, pube^a^, boys, girls	Resistance training, resistance program^a^, resistance intervention, resistance exercise, weight training, strength and conditioning	Weight, body composition, body mass, obese^a^, overweight, skinfold^a^, adiposity, waist, fat, dexa, BodPod

^a^Search term truncated

The screening and data extraction process was undertaken by HC and AD. Titles of potentially relevant articles were retrieved using the search strategy, duplicates then removed, and then the titles and abstracts were screened by HC. Ten percent (*n* = 418) of the titles and abstracts were also screened by AD. The inter-rater reliability for the two authors was found to be Kappa = 0.897 suggesting a strong level of agreement [16]. Full-text copies were obtained for potentially eligible articles and assessed for inclusion by HC and AD. During the review of full-text articles, a majority decision was taken in consultation with the other authors when disagreements regarding inclusion/exclusion occurred.

### The inclusion/exclusion criteria

Studies with participants of school age between 5 and 18 years were included. No studies were included where the subject group were identified as having a pathological condition or disability which affects movement, (e.g. cerebral palsy/dyspraxia) and no studies were included where the subject group was identified as having a behavioural or neuropsychological condition (e.g. autism or ADHD).

To allow an isolated review of strength training, studies included RT methods but were excluded if they contained plyometric, vibration or neuromuscular training, or training specifically for rehabilitation purposes. Additionally, studies were excluded if the warm up and cool down activities were more than 10 min duration each to ensure there was not a significant aerobic component in the intervention. Studies targeting physical activity or diet were not included unless both the control group and intervention group received the same dietary and physical activity intervention, but the intervention group received a different RT input. There was no restriction on location (e.g. school based or sports centre) or timing (e.g. during or after school).

Although studies were included that used a control group and also those that did not, for the purpose of this paper, the analysis focused on studies that included a control group, and therefore are referred to as controlled trials (CTs). Objective measures included a measure of weight status through body mass, body mass index (BMI), skinfolds, DEXA scans, BodPod, MRI scans, waist circumference, hip circumference or waist to hip ratio.

It is important to note that studies were only included when weight status was mentioned as an aim of the study in the abstract and not just as a demographic characteristic of the participants.

### Data extraction

Data were extracted using an electronic form by HC and included study characteristics (e.g. country, year); participant characteristics (e.g., age, sex); intervention components (e.g. setting, content); and changes in the outcomes (e.g. change in weight status). The outcome data were extracted in the form of mean, standard deviation and sample size. If the outcome data was not visible within the paper, authors were contacted and asked to provide this data within a set time frame. To check reliability, AD carried out data extraction on the first 10% of the included studies, which were in alphabetical order of the first author. Following this, any disagreements were resolved through discussion with all authors.

### Methodological quality and risk of bias assessment

The “Quality Assessment Tool for Quantitative Studies” developed by the Effective Public Health Practice Project in 1998 [17] was used to assess the quality and risk of bias of the included studies. The results of the assessment lead to an overall methodological rating of strong, moderate or weak in eight sections: selection bias, study design, confounders, blinding, data, collection methods, withdrawals and dropouts, intervention integrity and analysis. The assessment tool has been found to be valid and reliable [18]. To check reliability, AD carried out this assessment on 10% of the included studies and any disagreements were resolved through discussion between the two authors. Overall, the data extraction and risk of bias accuracy of one author was deemed to be acceptable.

### Meta-analysis

Random effects meta-analyses were conducted with the Comprehensive Meta-analysis software (version 2.2.064). Hedges’ *g* with randomised effects and 95% CIs were calculated for trials with sufficient data. The magnitude of Hedges’ *g* was interpreted using Cohen’s (1988) [19] convention as small (0.2), medium (0.5) and large (0.8). A significance level of *p* ≤ 0.05 was applied. Heterogeneity was assessed using the *I*^2^ statistic. For interpretation, *I*^2^ values of 25, 50 and 75 were considered to indicate low, moderate and high heterogeneity, respectively [20]. Publication bias was assessed by calculating Egger bias statistics [21] and Rosenthal’s fail-safe N [22]. Corresponding funnel plots were created.

A moderator analysis was conducted to determine whether the intervention effects on the outcomes differed by sex of participants (males or females), sex of training group (i.e. the training group was designed for either males, females or mixed sex), weight status (healthy weight, overweight, obese or mixed weight status), age (< 12 or > 12 years, based on primary and secondary school age split), pubertal stage (<Tanner stage 2 or >Tanner stage 2, based on pre-pubertal and post-pubertal stages), location (school during P.E, school during free time or community), type of control (no resistance training, nutrition input only, normal activity, wait list) and quality of study (weak, moderate or strong). Additional moderator analyses were planned for ethnicity and supervised compared to self-regulated sessions. However, there was insufficient data to allow these analyses. Although data was also extracted for frequency and duration of interventions, a moderator analysis was not conducted on this data due to the inappropriateness of separating their independent and combined impact on training outcomes.

It is important to note that for outcomes where a decrease in score was a positive intervention effect (e.g. BMI) and an increase in score was a positive effect (e.g. fat free mass), this was accounted for in the analysis.

## Results

Out of an initial 6290 studies identified through database searches, 18 studies met the inclusion criteria and included sufficient data for the meta-analysis. Twenty four data sets were included in the meta-analysis (some studies had more than one intervention group).

### Study characteristics

The surveyed studies were conducted in eight different countries (USA, Australia, New Zealand, Hong Kong, Brazil, Tunisia, Austria and Japan). In total, there were 554 participants in the experimental groups (sample sizes ranged from 8 to 78 participants) and 599 participants in the control groups (sample sizes ranging from 5 to 129 participants).

The age of all participants ranged from 8 to 16 years. Eight outcomes related to weight status were included in the analysis: body mass (kg), BMI (kg/m^2^), body fat (%), fat-free mass (kg), fat mass (kg), lean mass (kg), skinfolds (mm) and waist circumference (cm). The average attendance for the studies that reported it was 88%. The study details included in the analysis can be found in Table 2.

**Table 2:** Description of included studies/data sets

Study	Country	Participant details						Intervention details				Outcome measures	Quality score
		*N*	Age (years ± SD) or range	Pubertal stage (Tanner)	Weight status	% males	Sex of training group	Location	Weeks/× per week (min per session)	Sets/reps /intensity	Exercises		
Benson et al. 2008 [33]	New Zealand	EG 37	EG 12.3 ± 1.3	1–5	Mixed	59%	Mixed	Community	8 weeks/× 2	2 sets,8 reps, 80%1RM	Bicep screw curl, tricep extension, one arm dumbbell row, one dumbbell front raise, bench press, standing leg abduction, standing leg curl, calf raise, squat, abdominal crunch, abdominal reverse crunch.	Body mass (kg), body mass index (BMI), body fat (%), fat free mass (kg), fat mass (kg), waist circumference (cm)	2
		CG 41	CG 12.2 ± 1.3										
Chaouachi 2014 (a) [34]	Tunisia	EG 17	EG 11 ± 1	1–2	Healthy	100%	Males	Community	12 weeks/× 2	1–3 sets, 8–12 reps, /	Squats, lunges, alternate flat and incline chest press, unilateral shoulder flies or presses	Body mass (kg), body fat (%)	2
		CG 13	CG 11 ± 1										
Chaouachi et al. 2014 (b) [34]	Tunisia	EG 17	EG 11 ± 1	1–2	Healthy	100%	Males	Community	12 weeks/× 2	1–3 sets, 8–12 reps, Max weight, good form	Cleans, snatches, shoulder push press, kettlebell/dumbbell cross body pull.	Body mass (kg), body fat (%)	2
		CG 11	CG 11 ± 1										
Dos Santos Cunha et al. 2015 [35]	Brazil	EG 9	EG 10.4 ± 0.5	1	Healthy	100%	Males	School	12 weeks/× 3 (60 m)	3 sets, 6–15 reps, 60–80%1RM	Knee extension, elbow flexion, leg curl, bench press, hip adduction, inverse fly, and hip abduction	Body mass (kg), body fat (%), fat free mass (g), fat mass (g)	1
		CG 9	CG 10.9 ± 0.7										
Davis et al. 2009 [36]	USA	EG 17	EG 15.5 ± 1	4–5	O/W	/	/	Community	16 weeks/× 2 (60 m)	1–3 sets, 3–15 reps, 62–97% 1RM	Session 1 = leg press, deadlift, biceps curl, triceps extension, shoulder press. Session 2 = bench press, lat pull down, leg extension, leg curl, calf raises	Body mass (kg), body mass index (BMI), BMI percentile, BMI z score, fat mass (kg), lean mass (kg)	2
		CG 21	CG 15.5 ± 1										
Dorgo et al. 2009 [37]	USA	EG 63	EG 16 ± 1.2	/	Healthy	/	/	School	18 weeks/× 3 (80 m)	2–4 sets, 8–14 reps, partner resistance	Squat, seated chest press, bent-over row, seated row, lat pulldown, leg extension, overhead triceps extension, step lunge, shoulder press, seated fly, Romanian deadlift, good-morning, shoulder press, upright row, lying triceps extension, seated back extension, extended arm pressdown, standing biceps curl	Body mass index (BMI), skinfolds (mm)	2
		CG 129	CG 15.8 ± 1.1										
Faigenbaum et al. 1993 [38]	USA	EG 14	EG 10.8	1–2	Healthy	68%	Mixed	/	8 weeks/× 2 (35 m)	3 sets, 10–15 reps, 50–100%1RM	Leg extension, leg curl, overhead press, bicep curl, chest press	Body mass (kg), skinfolds (mm), waist circumference (cm)	2
		CG 10	CG 9.9										
Lau et al. 2004 [30]	Hong Kong	EG 21	10–17	/	Obese	68%	Mixed	Community	6 weeks/× 3(60 m)	3 sets > 5reps, 70–75%1RM	Chest press, lat pull down, shoulder press, leg press, leg extension, leg curl, heel raise, bicep curl, tricep extension, adjusted push-up	Body mass (kg), Body mass index (BMI), waist circumference (cm), hip circumference (cm), waist to hip ratio, lean mass (kg), fat mass (kg), body fat (%)	2
		CG 17											
Lillegaard et al. 1997 (a) [39]	USA	EG 20	EG 11.2	1–2	Healthy	100%	Mixed	/	12 weeks/× 3(60 m)	3 sets, 10 reps, 10RM	Barbell curl, tricep extension, leg press, leg curl, lat pulldown, bench press	Skinfolds (mm)	3
		CG 18	CG 10.1										
Lillegaard et al. 1997 (b) [39]	USA	EG 16	EG 14	3–5	Healthy	100%	Mixed	/	12 weeks/× 3(60 m)	3 sets, 10 reps, 10RM	Barbell curl, tricep extension, leg press, leg curl, lat pulldown, bench press	Skinfolds (mm)	3
		CG 10	CG 13.1										
Lillegaard et al., 1997 (c) [39]	USA	EG 8	EG 9.5	1–2	Healthy	0%	Mixed	/	12 weeks/× 3 (60 m)	3 sets, 10 reps, 10RM	Barbell curl, tricep extension, leg press, leg curl, lat pulldown, bench press	Skinfolds (mm)	3
		CG 6	CG 9.6										
Lillegaard et al. 1997 (d) [39]	USA	EG 8	EG 12.6	3–5	Healthy	0%	Mixed	/	12 weeks/× 3 (60 m)	3 sets, 10 reps, 10RM	Barbell curl, tricep extension, leg press, leg curl, lat pulldown, bench press	Skinfolds (mm)	3
		CG 5	CG 12.6										
Lubans et al. 2010 (a) [40]	Australia	EG 15	EG 14.9 ± 0.6	/	Mixed	0%	Mixed	School—free time	8 weeks/× 2 (50 m)	2 sets, 8–12 reps, max reps	Squat, lunge, calf raise, bent over row, bench press, front raise, biceps curl, triceps extension, crunch and Russian twist	Body mass (kg), waist circumference (cm), body mass index (BMI), fat mass (kg), fat free mass (kg), body fat (%)	2
		CG 16	CG 14.5 ± 0.6										
Lubans et al. 2010 (b) [40]	Australia	EG 22	EG 15.3 ± 0.8	/	Mixed	100%	Mixed	School—free time	8 weeks/× 2 (50 m)	2 sets, 8–12 reps, max reps	Squat, lunge, calf raise, bent over row, bench press, front raise, biceps curl, triceps extension, crunch and Russian twist	Body mass (kg), waist circumference (cm), body mass index (BMI), fat mass (kg), fat free mass (kg), body fat (%)	2
		CG 14	CG 14.8 ± 0.4										
Schranz et al. 2014 [41]	Australia	EG 30	EG 14.9 ± 1.4	2+	O/W/ obese	100%	Males	Community	24 weeks/× 3	1–3 sets, 8–12 reps, progress with good form	Bench press, leg press, lat pulldown, leg curl (lying or seated), shoulder press (seated), seated row, biceps curl, triceps pressdown, calf raise (seated) and abdominal crunch.	Body mass (kg), body mass index (BMI), skinfolds (mm), body fat (%), lean mass (kg)	1
		CG 26	CG 15.1 ± 1.6										
Schwingshandl et al. 1999 [42]	Austria	EG 14	EG 11 ± 2.5	/	Obese	43%	Mixed	Community	12 weeks/× 2 (60 m)	2 sets, 10 reps, 50–100%1RM	Lying leg press; seated leg extensions and leg curls; seated bench press; lat pulldown to the front and long pulley row; seated shoulder press; triceps pushdowns and triceps extension at the dips machine; dumbbell biceps curls; calf raises seated and crunches	Body mass (kg), body mass index (BMI) SDS, fat free mass (kg)	2
		CG 16	CG 12.2 ± 2.7										
Shaibi et al. 2006 [43]	USA	EG 11	EG 15.1 ± 0.5	3+	O/W	100%	Males	Community	16 weeks/× 2	1–3 sets, 3–15 reps, 52–97%1RM	session 1 = Leg press, dead lift biceps curl, triceps extension, shoulder press, session 2 = bench press, lat pull down, leg extension, leg curl calf raises	Body mass (kg), body mass index (BMI) percentile, fat mass (kg), lean mass (kg), body fat %	1
		CG 11	CG 15.6 ± 0.5										
Siegel et al. 1989 (a) [44]	USA	EG 26	EG 8.4	/	Healthy	100%	Mixed	School—free time	12 weeks/× 3 (30)	30 s work, 15 s rest, progress to 45 s work circuit training, body weights and weights up to 4.5lbs	Upper body self-supported locomotor movements (i.e., wheelbarrow, seal walk, crabwalk, etc.). A choreographed weight routine used tennis ball cans or detergent bottles filled with sand. Weights of 2.5, 3.0, 3.5, 4.0, and 4.5 lb. were offered. The third format was circuit training using various types of accessories as resistance, tennis balls for squeezing, and strips of rubber tire to pull	Body mass (kg), skinfolds (mm), body fat (%)	1
		CG 30	CG 8.6										
Siegel et al. 1989 (b) [44]	USA	EG 24	EG 8.7	/	Healthy	0%	Mixed	School—free time	12 weeks/× 3(30)	30 s work, 15 s rest, progress to 45 s work, circuit training, body weights and weights up to 4.5lbs	Upper body self-supported locomotor movements (i.e., wheelbarrow, seal walk, crabwalk, etc.). A choreographed weight routine used tennis ball cans or detergent bottles filled with sand. Weights of 2.5, 3.0, 3.5, 4.0, and 4.5 lb. were offered. The third format was circuit training using various types of accessories as resistance, tennis balls for squeezing, and strips of rubber tire to pull	Body mass (kg), skinfolds (mm), body fat (%)	1
		CG 16	CG 8.4										
Sigal et al. 2014 [29]	Canada	EG 78	EG 15.9 ± 1.5	4–5	Obese	31%	Mixed	Community	24 weeks/× 4	2–3 sets, 6–15 reps, moderate for 15 reps then 8RM	1: Bench press, chest fly lateral raise, shoulder, shrugs, bicep curl, tricep press, abdominal crunches. 2: Incline bench press, incline chest fly, shoulder press, front raise, preacher curl, assisted tricep dips, sit-ups. 3: Squat, leg curl, front lat pull down, seated row, lunge straight leg raise, abdominal crunches. 4: Leg press, leg extension, dumbbell pullover, seated row, lying knee extensions	Body mass (kg), body mass index (BMI), waist circumference (cm), body fat (%), fat mass (kg), lean mass (kg)	1
		CG 76	CG 15.6 ± 1.3										
Takai et al. 2013 [45]	Japan	EG 36	EG 13.6 ± 0.6	/	Healthy	100%	males	School—free time	8 weeks/× 4–6 (> 10 m)	100 reps, body weight	Squats	Body mass (kg), body mass index (BMI), body fat %, lean mass (kg)	1
		CG 58	CG 13.8 ± 0.5										
Treuth et al. 1998 [27]	USA	EG 11	EG 8.7 ± 0.7	1–2	Obese	0%	Females	School—free time	20 weeks/× 3 (20 m)	2 sets, 12–15 reps, 50%1RM	Bench press, military press, lat pull down, bicep curl, tricep extension, ab curls, leg press	Body mass (kg), body fat (%), fat mass (kg), lean mass (kg), fat free mass (kg)	1
		CG 11	CG 8.4 ± 0.9										
Velez et al. 2010 [46]	USA	EG 13	EG 16.1 ± 0.2	/	Mixed	57%	Mixed	School	12 weeks/× 3 (90 m)	2–3 sets, 10–15 reps, 80% 10RM	Bench press, seated row, shoulder press, lat pulldowns, flies, bicep curls, and tricep pushdowns or lower body exercises including squats, Romanian dead lift, leg extensions, leg curls, lunges, and calf raises.	Body fat (%), lean mass (kg), fat mass (kg), body mass index (BMI)	2
		CG 15											
Yoshimoto et al. 2016 [47]	Japan	EG 27	EG 13.8 ± 0.6	1–5	Healthy	0%	Females	School—free time	8 weeks/× 4–6 (> 10 m)	100 reps, body weight	Squats	Body mass (kg), Body mass index (BMI), body fat %, lean mass (kg)	1
		CG 20	CG 13.8 ± 0.5										

### Synthesis of results

**Fig. 2:**
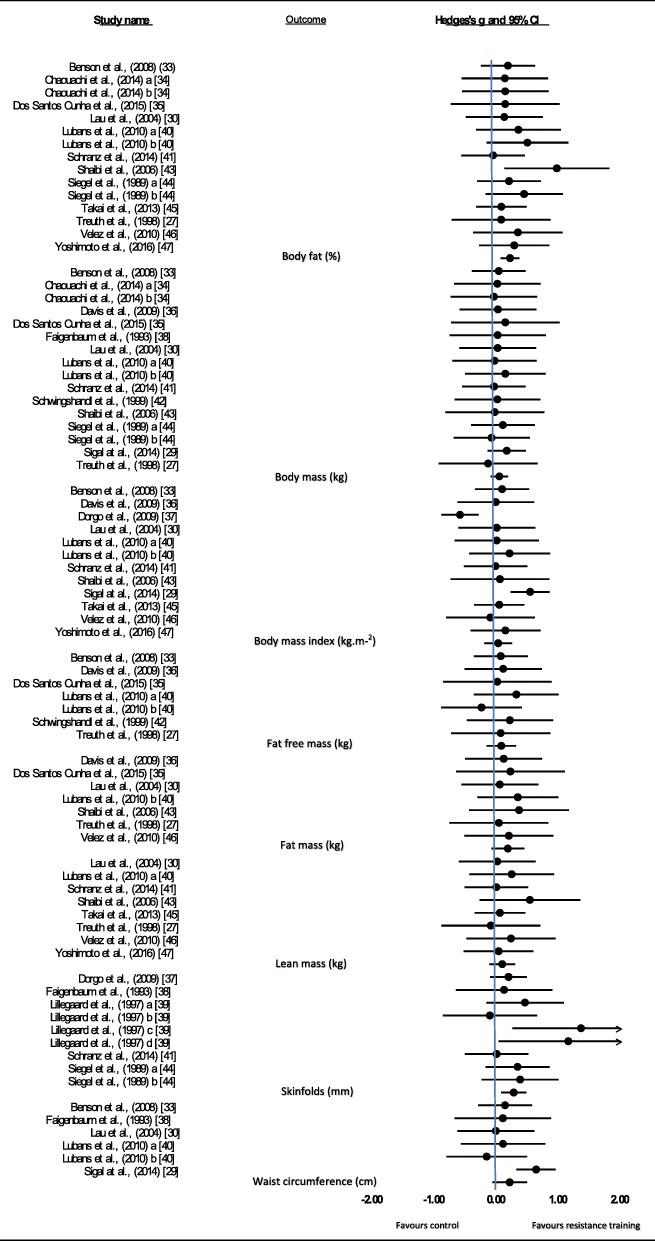
Summary of all weight status meta-analyses

For each study, Hedges’ *g* was calculated for each outcome variable to determine an overall intervention effect. Figure 2 illustrates effect sizes for all of the studies and the overall effect size for each outcome, which ranged from 0.024 to 0.274, indicating a trivial to small intervention effect relative to controls. Significant intervention effects were identified for body fat (%) (Hedges’ *g* = 0.215, 95% CI 0.059 to 0.371, *P* = 0.007) and skinfolds (mm) (Hedges’ *g* = 0.274, 95% CI 0.066 to 0.483, *P* = 0.01). Effect sizes were not significant for body mass (Hedges’ *g* = 0.043, 95% CI − 0.103 to 0.189, *P* = 0.564), body mass index (Hedges’ *g* = 0.024, 95% CI − 0.205 to 0.253, *P* = 0.838), fat-free mass (Hedges’ *g* = 0.073, 95% CI − 0.169 to 0.316, *P* = 0.554), fat mass (Hedges’ *g* = 0.180, 95% CI − 0.090 to 0.451, *P* = 0.192), lean mass (Hedges’ *g* = 0.089, 95% CI − 0.122 to 0.301, *P* = 0.408) or waist circumference (Hedges’ *g* = 0.209, 95% CI − 0.075 to 0.494, *P* = 0.149).

Based on the thresholds categorised [20], moderate to high heterogeneity was identified for BMI and waist circumference. For all other outcomes, heterogeneity was low.

### Publication bias

**Fig. 3:**
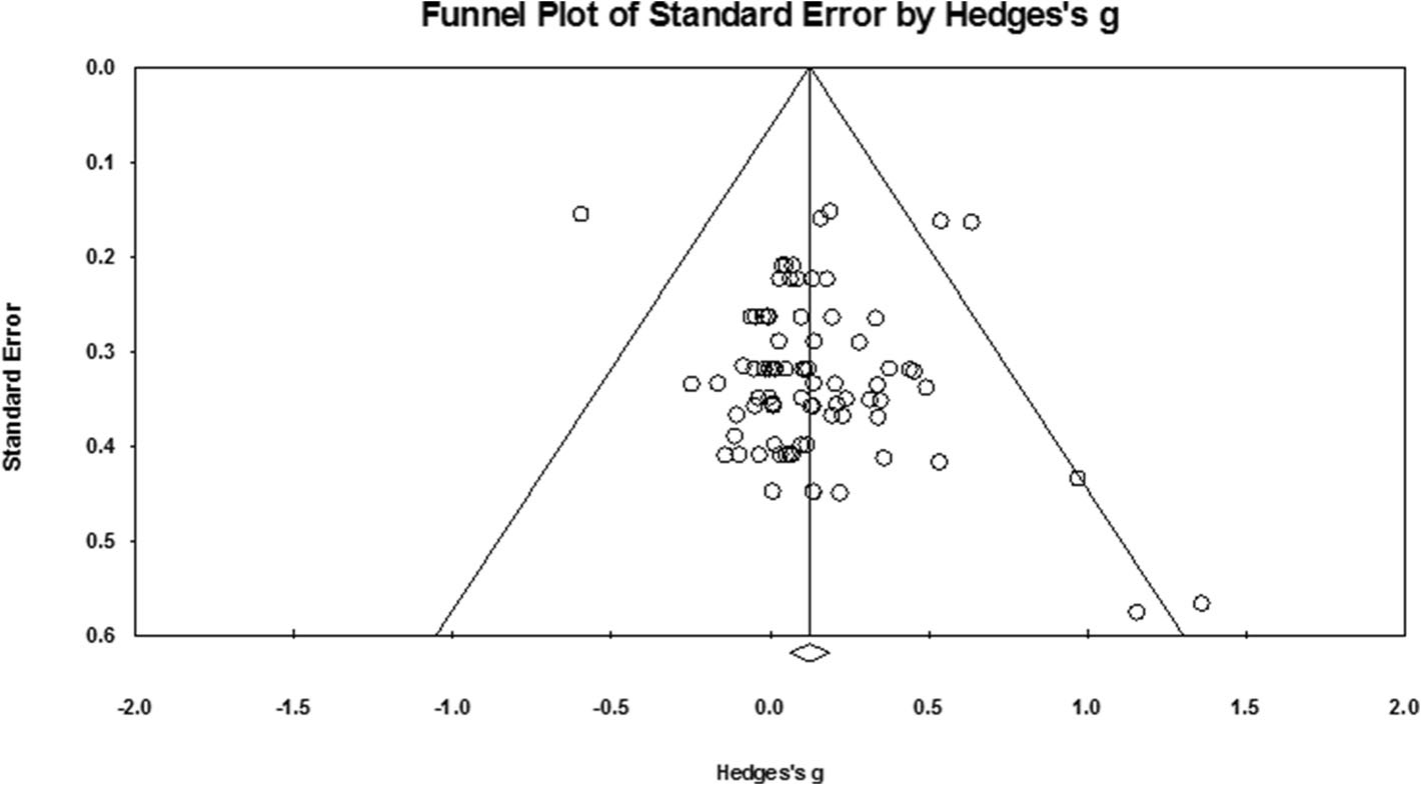
Funnel plot

To identify possible publication bias, effect sizes were plotted against standard errors to generate funnel plots as illustrated in Fig. 3. There was no indication of publication bias with no statistically significant result from Egger’s test [21]. Rosenthal’s fail-safe *N* [22] found that 241 additional studies would be needed for the cumulative effect to be non-significant. Therefore, it can be concluded that the meta-analysis provides a satisfactory representation of the effect of a RT intervention on weight status.

### Quality appraisal

Through the quality appraisal process, 44% of the included studies were classified as ‘strong’, 50% were classified as ‘moderate’ and 6% were classified as ‘weak’.

### Moderator analysis

The results (Table 3) suggest a moderation effect of weight status on BMI and waist circumference, with significant differences between healthy weight, mixed weight, overweight/obese and obese participants. The results suggest a moderation effect of quality score on waist circumference, with significant differences between strong and moderate studies.

**Table 3:** Moderator analysis

Moderator	Outcome	Hedges’*g* (95% CI)	No. of studies	Between group comparison: *Q* (df)
Sex of participants	Skinfolds (mm)	Females = 0.815 (0.167 to 1.463)*	3	3.310 (2)
		Males = 0.188 (− 0.105 to 0.482)	4	
		Mixed = 0.181 (− 0.100 to 0.462)	2	
Sex of training group	Body fat (%)	Mixed = 0.277 (0.055 to 0.500)*	7	0.660 (2)
		Males = 0.139 (0.− 0.108 to 0.387)	6	
		Females = 0.212 (− 0.254 to 0.678)	2	
	Skinfolds (mm)	Mixed = 0.320 (0.094 to 0.545)**	8	1.214 (1)
		Males = − 0.001 (− 0.519 to 0.517)	1	
Weight status	Body mass index (kg/m^2^)	Obese = 0.540 (0.220 to 0.860)**	1	8.586 (3)*
		Healthy = − 0.172 (− 0.668 to 0.324)	3	
		Mixed = 0.063 (−0.231 to 0.358)	4	
		Overweight/obese = −0.003 (− 0.313 to 0.307)	4	
	Body fat (%)	Mixed = 0.301 (0.005 to 0.598) *	4	0.374(2)
		Healthy = 0.189 (− 0.032 to 0.410)	7	
		Overweight/obese = 0.198 (− 0.203 to 0.598)	4	
	Skinfolds (mm)	Healthy = 0.320 (0.094 to 0.545)**	8	1.241 (1)
		Overweight/obese = − 0.001 (− 0.519 to 0.517)	1	
	Waist circumference (cm)	Obese = 0.636 (0.314 to 0.958)***	1	7.586(3)*
		Healthy = 0.100 (− 0.684 to 0.884)	1	
		Mixed = 0.056 (− 0.267 to 0.379)	3	
		Overweight/obese = −0.015 (0.083 to 0.497)	1	
Age	Body fat (%)	> 12 = 0.211 (0.016 to 0.407)*	8	0.004(1)
		< 12 0.222 (− 0.036 to 0.480)	7	
	Skinfolds (mm)	< 12 = 0.415 (0.116 to 0.714) **	5	1.294 (1)
		> 12 = 0.165 (− 0.146 to 0.476)	4	
Pubertal stage	Skinfolds (mm)	< 2 = 0.415 (0.116 to 0.714) **	5	0.392 (1)
		> 2 0.199 (− 0.7407 to 0.805)	3	
	Waist circumference (cm)	> 2 = 0.636 (0.314 to 0.958) ***	1	0.536(1)
		< 2 = 0.100 (− 0.684 to 0.884)	1	
Location	Body mass index (kg/m^2^)	School free time = 0.277 (0.069 to 0.485)*	5	2.152(2)
		School PE = − 0.103 (− 0.825 to 0.618)	1	
		Community = 0.027 (− 0.227 to 0.280)	5	
	Body fat (%)	School free time = 0.256 (0.030 to 0.483)*	6	0.337(2)
		School PE = 0.259 (− 0.301 to 0.820)	2	
		Community = 0.164 (− 0.069 to 0.397)	7	
Quality score	Body fat (%)	Moderate = 0.269 (0.001 to 0.537)*	5	0.282 (2)
		Weak = 0.135 (− 0.363 to 0.632)	2	
		Strong = 0.197 (− 0.011 to 0.405)	8	
	Waist circumference (cm)	Strong = 0.636 (0.314 to 0.958)**	1	7.528 (1)**
		Moderate = 0.048 (− 0.221 to 0.317)	5	

*p < 0.05, **p < 0.01, ***p < 0.001

## Discussion

### Summary of evidence

The purpose of this meta-analysis was to examine the effect of resistance training (RT) interventions on weight status in youth. In summary, there was a small, statistically significant, effect of RT interventions on body fat % and skinfolds, but no overall significant effect on body mass, BMI, fat-free mass, fat mass, lean mass or waist circumference.

The UKSCA’s [8] and NSCA’s [7] position statements on youth RT both suggest that RT may have a positive impact on body fat and the significant findings of this meta-analysis for body fat % and skinfolds support these statements.

While more studies are required to provide further understanding of the mechanism underlying a reduction in body fat due to a RT intervention, it has been reported that a possible cause could be due to an increase in skeletal muscle mass and resulting increase in basal metabolic rate [23], in particular, this has been noted in adolescents [8]. However, as there were no overall significant effect sizes evident for lean mass or fat free mass in this analysis, this suggests that these changes could possibly be due to increases in overall energy expenditure that may have occurred by simply taking part in an active intervention, rather than an increase in metabolically active lean tissue. It is important to note, however, that with data from both pre-pubertal and post-pubertal participants included in the analysis, this may have had an impact on the results.

Although all of the studies that measured fat mass were in favour of the intervention, this result was not significant and the effect size was trivial. It is worth considering why the effects were not consistent across different measures of body fat. Although body scans have been used in the included studies to measure both body fat % and fat mass, studies that have used skinfolds to measure body fat % have also been included in the analysis. DEXA scans measure both subcutaneous and visceral fat; however, skinfolds measure subcutaneous fat only. As there have been variable findings with regards to the impact of training on different locations of fat [24–28], this could explain why there were significant findings for body fat % and skinfolds, but not for fat mass. This emphasises the importance of validity when selecting measurement tools and methods used to assess intervention outcomes indicative of body fat. An additional factor to consider in this meta-analysis is that there was more than double the number of studies that investigated body fat % as an outcome in comparison to fat mass, providing greater statistical power to the findings for body fat %.

Resistance training did not demonstrate a significant effect on body mass or BMI; however, due to growth, these results should be interpreted with caution. Since the studies included in the analysis assessed a combination of healthy weight, overweight and obese participants, changes in these measures over time are likely to be variable and this is explored further in the moderator analysis discussed below. For the purpose of this analysis, a decrease in body mass and BMI was analysed as a favourable change. However, with the interventions being resistance based, a subsequent increase in lean mass/fat-free mass (and therefore body mass) was possible, and this may have obscured the findings with an overall positive trend for RT impacting on lean mass and fat free mass in this analysis. It has been identified that there is mixed evidence with regards to whether youth may experience increases in muscle mass following RT, most likely due to inadequate levels of circulating testosterone [7] although it has been suggested that resistance training may develop lean body mass in adolescents [8]. This will have been exacerbated by the inclusion of youth from 8 to 16 years of age of varying pubertal status and by varying intervention duration. It has also been suggested that periods of training in excess of 10 weeks are required for increases in lean muscle mass to occur [10]. In this review, for lean mass, 67% of the data sets were from studies that included interventions that were > 10 weeks, and for fat-free mass, these interventions were > 10 weeks for only 50% of the studies. This suggests that the intervention duration for several of the studies may not have been long enough to invoke positive measurable changes.

For the outcome of waist circumference, there was no significant effect size evident. With a combination of healthy weight, overweight and obese participants, it might be expected that those studies including overweight/obese participants would show a larger effect on waist circumference than the studies that included healthy weight participants, and this is further explained by the moderator analysis discussed below. There were two studies that included obese participants only; one finding a large effect of the intervention on waist circumference [29] and the other finding no significant effect [30]. In the 2004 study, the authors did identify that with only a 6-week intervention, this may not have been long enough to have a positive impact on the measured outcomes. Additionally, only six studies investigated waist circumference as an outcome, and this outcome therefore had less statistical power than some of the others.

### Previous reviews

Overall, these findings are similar to previous reviews. In a meta-analysis published in 2013 [12], there was a significant effect of interventions including a RT component on body fat % in overweight or obese youths. They also reported no significant effect sizes for body mass, BMI waist circumference, fat mass or lean mass. Out of the 40 studies included, only 9 studies were RT-only studies, and out of these 9, 6 were CTs. However, although interventions were included that also incorporated an aerobic and/or dietary component, similar effects were found for studies that included interventions that were RT alone when authors performed a sub-analysis. There were only three studies included that incorporated RT only and therefore interpreting the data should be undertaken with caution.

In a further systematic review [11], only six studies were included that investigated RT only interventions in overweight/obese youth. Three out of the six studies showed a significant decrease in percentage fat and a significant increase in fat-free mass, although none of the studies found a decrease in total fat mass, which is in support of the findings of this review. Four studies reported significant changes in body composition, with an increase in fat-free mass and BMI and additionally, all studies reported an increase in body weight. This is conflicting with the current review, although their review only included overweight and obese youth. Unlike the studies in the current analysis, all studies in their review included RT of moderate to submaximal intensity during treatment, rather than high intensity work and while acknowledging the limitations of using percentage of 1RM to prescribe intensity, higher intensity work (however calculated) will provide a greater stimulus for overload than low-medium intensity work. This may have had an impact on their overall findings with regards to weight status, as high intensity work has been reported as a key component to elicit changes in body composition [10]. Supporting the variable findings with regards to the impact of RT on obese adolescents, inconclusive results were found from just seven studies included that focused on RT alone [10].

In the only systematic review to date that included both healthy weight and overweight/obese participants, 12 studies were included [14]. It was reported that for CTs, no studies found a significant change in BMI body fat, two studies reported a change in lean body mass and one study that reported a change in waist circumference. In their review, there were only five RCTs and three of these included a dietary and/or aerobic component. The interventions were also different to the current review with all but two of the RT interventions being circuit based.

Overall, with just one of the reviews described above including a meta-analysis, it makes comparisons with the current meta-analysis somewhat challenging.

### Moderator analysis

To investigate the findings further, a moderator analysis was completed on all outcomes to identify if any effects could be explained by specific moderator variables. It was found that weight status was a moderator for BMI and waist circumference, and these outcomes showed moderate and high heterogeneity respectively and therefore the variance between studies could be explained by weight status. All other outcomes did not display significant heterogeneity, and no significant findings were apparent from the moderator analysis (Table 2).

There was a significant effect of the intervention on the BMI and waist circumference of obese participants (Table 2) but not on other weight categories, indicating that RT could be an effective intervention on these outcomes in obese individuals. It would seem plausible that BMI and waist circumference outcomes would vary significantly across studies due to the inclusion of both healthy weight and overweight/obese participants. It has been reported that obese youth are more sedentary than their healthy weight peers [31] and require more energy to move [32]. Therefore, an increase in physical activity might have a larger relative increase in energy expenditure reflected in reduced BMI and waist circumference. It should be noted, however, that for waist circumference, there were only six studies included in the analysis. Three were mixed weight with one study each for obese, overweight and healthy weight participants. This small number of studies may explain the high heterogeneity, and therefore interpreting the results should be undertaken cautiously. Additionally, a longer term follow up study would be beneficial to investigate resistance training as an obesity prevention method.

These findings conflict with previous findings [12] that suggested that there was a very small moderation effect of age and sex on various weight status outcomes. It was reported that for youth 12 years or older, there was an intervention effect on body mass, BMI, waist circumference, body fat %, fat mass and lean mass, and for males, there was an intervention effect on body mass, BMI, body fat % and fat mass. However, these were small influences on intervention effects, and their analysis included studies that incorporated an aerobic or dietary component which is different to the analysis in the current review and therefore it is difficult to make conclusions regarding RT alone based on their findings.

### Strengths and limitations

There were a number of strengths to this review. There should be strong confidence in the main findings given the rigorous review process. A strict inclusion/exclusion criteria resulted in an analysis of 24 data sets that examined the effects of RT on weight status in 554 youths from 8 countries.

This is also the first meta-analysis to include healthy weight and overweight/obese participants taking part in RT only interventions, which is important to identify the impact of RT not only as a treatment for obesity but also as a prevention.

There was high compliance reported in the included studies. For the studies who reported it, compliance was 88%. As well as a strength of the current meta-analysis, high compliance adds substance to the potential for RT as a viable mode of intervention to improve weight status.

There are however limitations that need to be considered when interpreting the results. There was large variability within the study interventions with regards to participant numbers (ranging from 5 to 129 participants), frequency, duration and programme content. The frequency ranged from 2 to 6 times a week and duration ranged from 8 to 20 weeks. Programmes also involved a mixture of sets and reps with a range of intensities and some being circuit based. The forest plots also indicate large variation with the individual studies’ results.

For certain outcomes there were a variety of different methods of measurement. For example, body fat % was measured by DEXA, BodPod, bioelectrical impedance scales, skinfolds and MRI scanning. While reporting standardised mean differences allowed us to pool the data for the purposes of the meta-analysis, differences between measurement tools should be acknowledged.

A limitation of the moderator analysis was not all of the studies reported data to enable a thorough investigation, so limited conclusions can be made based on this additional, incomplete level of analysis.

Finally, there was a mixture of quality of the studies included, with only 44% of the studies classified as ‘strong’. When moderating for quality of studies, it is difficult to make conclusions despite there being significant findings for waist circumference, as there was only one ‘strong’ study for this outcome. This had a large effect size in comparison to the ‘moderate’ and ‘weak’ studies.

## Conclusions

The literature suggests that RT may have a positive effect on weight status in youth, although the effects have not been clearly established and it is clear that more quality research is required to investigate this further. This meta-analysis provides an overview of the current research evidence and an insight into the potential benefits of such interventions. It adds to previous systematic reviews by including both healthy weight and overweight/obese youths and including RT only interventions which explores not only the treatment of obesity, but also prevention which is a vital component in combatting rising levels of obesity in children.

Overall, this meta-analysis found a small, statistically significant effect of RT interventions on body fat % and skinfolds, but no overall significant effect on body mass, BMI, fat free mass, fat mass, lean mass or waist circumference. While we can conclude that RT interventions have a small positive impact on some indicators of weight status, it is noted that this reflects only a small body of published work.

With RT interventions offering potential benefits for youth with regards to weight status, it is imperative that more robust and quality studies should be conducted to further, and unequivocally, investigate the role RT may play in the treatment and prevention of obesity. Based on the findings of this meta-analysis, and in support of the conclusions of previous reviews, future studies should be designed as randomised controlled trials with large samples and include a treatment group with an isolated RT intervention. There should be careful consideration into appropriate intervention content and assessment methods. If validated, this type of intervention, as recommended by the UK and WHO physical activity guidelines, could ultimately have a positive impact on the prevalence of common obesity-related diseases, such as type 2 diabetes, cardiovascular disease and cancer, and improve the health of individuals not only during childhood but as they progress through life.

## References

[1] World Health Organization. Obesity and overweight. 2016. Available from: http://www.who.int/mediacentre/factsheets/fs311/en/ Accessed 09/11/17.

[2] Ogden CL, Carroll MD, Curtin LR (2006). Prevalence of overweight and obesity in the United States, 1999-2004. JAMA.

[3] World Health Organization (2010). Global Recommendations on Physical Activity for Health.

[4] Chief Medical Office. UK Physical Activity Guidelines. 2011. Available from: https://www.gov.uk/government/publications/uk-physical-activity-guidelines Accessed 1.10.17.

[5] Health Behaviour in School-aged Children (HBSC) survey. Physical activity in adolescents. 2013/14. Available from: http://www.euro.who.int/__data/assets/pdf_file/0018/303480/HBSC-No.7_factsheet_Physical.pdf?ua=1 Accessed 09/11/17.

[6] Van Sluijs EM, McMinn AM, Griffin SJ (2007). Effectiveness of interventions to promote physical activity in children and adolescents: systematic review of controlled trials. BMJ.

[7] Faigenbaum AD, Kraemer WJ, Blimkie CJR (2009). Youth resistance training: updated position statement paper from the national strength and conditioning association. J Strength Cond Res..

[8] Lloyd RS, Faigenbaum AD, Myer GD (2012). UKSCA position statement: youth resistance training. Prof Strength Cond.

[9] Stratton G, Jones M, Fox KR (2004). BASES position statement on guidelines for resistance exercise in young people. J Sports Sci.

[10] Alberga AS, Sigal RJ, Kenny GP (2011). A review of resistance exercise training in obese adolescents. Phys Sportsmed.

[11] Dietz P, Hoffmann S, Lachtermann E, Simon P (2012). Influence of exclusive resistance training on body composition and cardiovascular risk factors in overweight or obese children: a systematic review. Obesity Facts.

[12] Schranz N, Tomkinson G, Olds T (2013). What is the effect of resistance training on the strength, body composition and psychosocial status of overweight and obese children and adolescents? A systematic review and meta-analysis. Sports Med.

[13] Pandita A, Sharma D, Pandita D (2016). Childhood obesity: prevention is better than cure. Diabetes Metab Syndr Obes.

[14] Benson AC, Torode ME, Fiatarone Singh MA (2008). Effects of resistance training on metabolic fitness in children and adolescents: a systematic review. Obes Rev.

[15] Moher D, Liberati A, Tetzlaff J, Altman DG (2009). Preferred reporting items for systematic reviews and meta-analyses: the PRISMA statement. Br Med J.

[16] McHugh L (2012). Interrater reliability: the kappa statistic. Biochem Med.

[17] Effective Public Health Practice Project. Quality Assessment Tool For Quantitative Studies. 1998. Available from: https://merst.ca/wp-content/uploads/2018/02/quality-assessment-tool_2010.pdf. Accessed 09 Aug 2018.

[18] Thomas BH, Ciliska D, Dobbins M (2004). A process for systematically reviewing the literature: providing the research evidence for public health nursing interventions. Worldviews Evid-Based Nurs.

[19] Cohen J (1988). Statistical power analysis for the behavioral sciences.

[20] Higgins JP, Thompson SG, Deeks JJ (2003). Measuring inconsistency in meta-analyses. British Med J.

[21] Egger M, Davey Smith G, Schneider M (1997). Bias in meta-analysis detected by a simple, graphical test. BMJ.

[22] Rosenthal R (1979). The file drawer problem and tolerance for null results. Psychol Bull.

[23] Smith JJ, Eather N, Morgan PJ (2014). The health benefits of muscular fitness for children and adolescents: a systematic review and meta-analysis. Sports Med.

[24] Slentz CA, Bateman LA, Willis LH (2011). Effects of aerobic vs. resistance training on visceral and liver fat stores, liver enzymes, and insulin resistance by HOMA in overweight adults from STRRIDE AT/RT. Am J Physiol Endocrinol Metab.

[25] Lee S, Deldin AR, White D (2013). Aerobic exercise but not resistance exercise reduces intrahepatic lipid content and visceral fat and improves insulin sensitivity in obese adolescent girls: a randomized controlled trial. Am J Physiol Endocrinol Metab.

[26] Lee S, Bacha F, Hannon T (2012). Effects of aerobic versus resistance exercise without caloric restriction on abdominal fat, intrahepatic lipid, and insulin sensitivity in obese adolescent boys a randomized, controlled trial. Diabetes.

[27] Treuth MS, Hunter GR, Figueroa-Colon R (1998). Effects of strength training on intra-abdominal adipose tissue in obese prepubertal girls. Med Sci Sports Exerc.

[28] Kordi R, Dehghani S, Noormohammadpour P (2015). Effect of abdominal resistance exercise on abdominal subcutaneous fat of obese women: a randomized controlled trail using ultrasound imaging assessments. J Manip Physiol Ther.

[29] Sigal RJ, Alberga AS, Goldfield GS (2014). Effects of aerobic training, resistance training, or both on percentage body fat and cardiometabolic risk markers in obese adolescents: the healthy eating aerobic and resistance training in youth randomized clinical trial. JAMA Pediatr.

[30] Lau PWC, Yu CW, Lee A (2004). The physiological and psychological effects of resistance training on Chinese obese adolescents. J Exerc Sci Fit.

[31] Ebbeling CB, Pawlak DB, Ludwig DS (2002). Childhood obesity: public-health crisis, common sense cure. Lancet.

[32] Maffeis C, Schutz Y, Schena F (1993). Energy expenditure during walking and running in obese and nonobese prepubertal children. J Pediatr.

[33] Benson AC, Torode ME, Fiatarone (2008). The effect of high-intensity progressive resistance training on adiposity in children: a randomized controlled trial. Int J Obes.

[34] Chaouachi A, Hammami R, Kaabi S (2014). Olympic weightlifting and plyometric training with children provides similar or greater performance improvements than traditional resistance training. J Strength Cond Res.

[35] Dos Santos CG, Sant'anna MM, Cadore EL (2015). Physiological adaptations to resistance training in prepubertal boys. Res Q Exerc Sport.

[36] Davis JN, Kelly LA, Lane CJ (2009). Randomized control trial to improve adiposity and insulin resistance in overweight Latino adolescents. Obesity.

[37] Dorgo S, King GA, Candelaria NG (2009). Effects of manual resistance training on fitness in adolescents. J Strength Cond Res..

[38] Faigenbaum AD, Zaichkowsky LD, Westcott WL (1993). The effects of a twice-a-week strength training program on children. Pediatr Exerc Sci.

[39] Lillegaard WA, Brown EW, Wilson DJ (1997). Efficacy of strength training in prepubescent to early postpubescent males and females: effects of gender and maturity. Pediatr Rehabil.

[40] Lubans DR, Sheaman C, Callister R (2010). Exercise adherence and intervention effects of two school-based resistance training programs for adolescents. Prev Med.

[41] Schranz N, Tomkinson G, Parletta N (2014). Can resistance training change the strength, body composition and self-concept of overweight and obese adolescent males? A randomised controlled trial. Br J Sports Med.

[42] Schwingshandl J, Sudi K, Eibl B (1999). Effect of an individualised training programme during weight reduction on body composition: a randomised trial. Arch Dis Child.

[43] Shaibi GQ, Cruz ML, Ball GD (2006). Effects of resistance training on insulin sensitivity in overweight Latino adolescent males. Med Sci Sports Exerc.

[44] Siegel JA, Camaione DN, Manfredi TG (1989). The effects of upper body resistance training on prepubescent children. Pediatr Exerc Sci.

[45] Takai Y, Fukunaga Y, Fujita E (2013). Effects of body mass-based squat training in adolescent boys. J Sports Sci Med.

[46] Velez A, Golem DL, Arent SM (2010). The impact of a 12-week resistance training program on strength, body composition, and self-concept of Hispanic adolescents. J Strength Cond Res.

[47] Yoshimoto T, Takai Y, Fukunaga Y (2016). Effects of school-based squat training in adolescent girls. J Sports Med Phys Fitness.

